# Microplastics in urban environments: The dog as a bioindicator of exposure and human gastrointestinal risk

**DOI:** 10.7705/biomedica.8117

**Published:** 2026-08-20

**Authors:** Galo Martínez-Cepeda, Daniel Ramos-Sarmiento, Gianina Sailema-Rosado, Allisson Sangache-Guamán, Natalia Bustos-Chávez, Holger Salcán-Guamán, Mario Fernández-Morales, Linda Díaz-Cevallos

**Affiliations:** 1 Facultad de Medicina Veterinaria y Agronomía, Universidad UTE, Santo Domingo, Ecuador Facultad de Medicina Veterinaria y Agronomía Universidad UTE Santo Domingo Ecuador; 2 Profesionales independientes, Guayaquil, Ecuador Profesionales independientes Guayaquil Ecuador; 3 Plataforma de Microscopía Electrónica, Instituto Nacional de Investigación en Salud Pública Dr. Leopoldo Izquieta Pérez, Guayaquil, Ecuador Instituto Nacional de Investigación en Salud Pública Dr. Leopoldo Izquieta Pérez Guayaquil Ecuador

**Keywords:** Microplastics, gastrointestinal diseases, one health, sentinel species, humans, dogs, Ecuador, microplásticos, enfermedades gastrointestinales, salud única, especies centinela, humanos, perros, Ecuador

## Abstract

**Introduction.:**

Microplastics, particles smaller than 5 mm, are an environmental and public health concern due to their widespread presence in domestic environments. They affect humans and animals that share water, food, and environments. In Santo Domingo (Ecuador), companion dogs can act as environmental sentinels, reflecting exposure to these contaminants and their potential association to health problems.

**Objective.:**

To evaluate microplastics in dog feces in Santo Domingo and their possible relationship with domestic plastic waste and gastrointestinal diseases in their human tutors.

**Materials and methods.:**

Feces from 27 domestic dogs were analyzed using potassium hydroxide (KOH) digestion and microscopy to identify and quantify microplastics. Daily production of plastic waste in contact with food and the presence of gastrointestinal diseases in tutors were recorded. Statistical analyses were applied to evaluate correlations and associations, considering a significance level of α = 0.05.

**Results.:**

Fecal samples from 21 dogs contained microplastic particles, with an average of 288.89 microplastic particles per gram, predominantly plastic fibers. The northeastern and northwestern areas showed the highest levels. No significant correlation was found between plastic waste generation and microplastics load (p = 0.194), but a significant association was found with gastrointestinal diseases in tutors (p = 0.027; Cramer's V = 0.463).

**Conclusions.:**

Dogs reflect domestic exposure to microplastics, and its study provides evidence of shared risks to both human and animal health. The need to control plastic pollution and promote inter-sectorial preventive research and actions under the *One Health* approach is reinforced.

## Resumen

**Introducción.:**

Los microplásticos, fragmentos menores de 5 mm, son un problema ambiental y sanitario por su amplia presencia en los entornos domésticos. Afectan humanos y animales que comparten agua, alimentos y espacios. En Santo Domingo (Ecuador), los perros de compañía pueden actuar como centinelas ambientales, reflejando la exposición a estos contaminantes y su posible relación con diversos problemas de salud.

**Objetivo.:**

Evaluar los microplásticos en las heces de perros de Santo Domingo y su posible relación con residuos plásticos domésticos y con enfermedades gastrointestinales de sus tutores.

**Materiales y métodos.:**

Se analizaron las heces de 27 perros domésticos mediante digestión con hidróxido de potasio (KOH) y microscopía para identificar y cuantificar los microplásticos. Se registró la generación diaria de residuos plásticos en contacto con alimentos y la presencia de enfermedades gastrointestinales de los tutores. Se aplicaron análisis estadísticos para evaluar correlaciones y asociaciones, con α = 0,05 como nivel significativo.

**Resultados.:**

Ventiún muestras fecales contenían microplásticos, con una media de 288,89 partículas de microplásticos por gramo, con predominio de fibras plásticas. Las zonas noreste y noroeste fueron las más afectadas. No se halló correlación significativa entre la generación de residuos plásticos y la carga de microplásticos (p = 0,194), pero sí una asociación significativa con enfermedades gastrointestinales de los tutores (p = 0,027; V de Cramer = 0,463).

**Conclusiones.:**

Los perros reflejan la exposición doméstica a microplásticos y su estudio aporta evidencia sobre los riesgos compartidos para la salud humana y animal. Se refuerza la necesidad de controlar la contaminación plástica y fomentar investigaciones y acciones preventivas intersectoriales bajo el enfoque *One Health.*

Microplastics, defined as plastic particles smaller than 5 mm, and nanoplastics, < 0.1 μm, represent an emerging environmental and public health threat due to their widespread presence in water, soil, and food ^1^^-^^5^. These contaminants originate from the degradation of larger plastic materials or from manufactured products such as cosmetics and cleaning agents ^6^^,^^7^. They occur in various forms (fibers, spheres, or fragments) which complicate their detection and regulation ^8^.

Biologically, microplastics can act as vectors for toxic substances such as heavy metals and pesticides, which accumulate in tissues and induce oxidative stress, cellular damage, and metabolic alterations ^2^^,^^9^^-^^11^. Their ingestion has been associated with digestive disorders, nutritional deficiencies, and reproductive impairments ^12^^,^^13^.

Microplastics have been detected in human tissues, demonstrating direct exposure ^11^^,^^14^, and in domestic animals that share similar environmental conditions and risks with humans ^15^^-^^17^. In dogs, microplastic ingestion has been associated with intestinal inflammation, microbiota imbalance, and chronic digestive symptoms ^18^^-^^20^, as well as potential hepatic, renal, and endocrine disruptions ^21^^,^^22^. Microplastics may also be inhaled through household dust, contributing to respiratory irritation and dysfunction ^23^.

Within this context, the present study evaluated the presence of microplastic particles in fecal samples from domestic dogs and explored their possible association with gastrointestinal disorders in their human tutors. These preliminary findings provide evidence of shared microplastic exposure in urban environments, contributing to a broader understanding of environmental and health risks under the *One Health* approach.

## Materials and methods

### Study group description

In this exploratory cross-sectional analytical study, potential associations between microplastics exposure and digestive health in dogs and humans sharing a common domestic environment were evaluated. To this end, the presence of microplastic particles was examined in fecal samples from 27 dogs belonging to households located in urban and peri-urban areas of the Santo Domingo canton, in the province of Santo Domingo de los Tsáchilas, Ecuador. Household selection was conducted by convenience and randomized across the entire canton.

According to information provided by the tutors, the dogs received two to three daily portions of mixed food (a combination of commercial and homemade diets) and had *ad libitum* access to water. Simultaneously, the daily consumption of food-contact plastic products by human family members was estimated, and self-reported gastrointestinal diseases were recorded.

No selection criteria were applied based on age, breed, or sex, as the study aimed to identify the presence of microplastic particles at the species level without focusing on specific demographic variables, given that this contaminant may affect any living organism indiscriminately.

### Sample collection

Approximately 5 g of feces per animal were collected and individually stored in glass jars with screw caps, properly labeled for identification. Samples were then transported under a cold chain to the multifunctional laboratories of *Universidad Tecnológica Equinoccial (UTE),* Santo Domingo campus, for further analysis.

### Microplastics digestion and flotation

A modified version of the Willis-Molloy method was used, adapted for microplastic particles recovery ^5^^,^^24^^,^^25^, dissolving 1 g of fecal sample in 10 ml of 10% potassium hydroxide (KOH) to digest organic material. This mixture was incubated for 72 hours at a constant temperature of 60 °C. In addition, positive controls consisting of polyethylene fibers and fragments, as well as blank samples without fecal material, were prepared and subjected to the same chemical treatment and identical incubation and handling conditions. Subsequently, 1 ml of the supernatant was extracted for observations under conventional optical microscopy and scanning electron microscopy (SEM).

### Optical microscopy and scanning electron microscopy analysis

A 50 Lil aliquot of the supernatant was placed on a slide. After a 5-minute rest to allow particle sedimentation, samples were systematically examined using the smear technique. Quantification of microplastic polymers was performed with an Olympus™ CX-31 binocular microscope, employing a 40X objective.

For morphological characterization, samples were prepared for SEM analysis. They were first air-dried for 4 hours and then placed in an oven at 100 °C for 6 hours. Subsequently, they were transferred to the *Instituto Nacional de Investigación en Salud Pública* "Dr. Leopoldo Izquieta Pérez" in Guayaquil, where an additional 3 hour drying at 150 °C was performed.

A thin gold coating was applied using a JEOL™ JPC-1200 device. Finally, images were captured with a JEOL™ JSM-IT500 scanning electron microscope, operated with its proprietary JSM IT500, version 1.300, software.

### Statistical analysis

Collected data were initially subjected to exploration analysis and normality tests (Shapiro-Wilk) to determine variable distribution and the appropriateness of parametric or non-parametric tests.

To assess associations between categorical variables, chi square (x^2^) independence tests were applied. For quantitative variables, Spearman correlation coefficients were calculated according to data distribution.

All analyses were conducted with a statistical significance level of α = 0.05. Data processing and analysis were performed using IBM SPSS™ Statistics (version 25) and InfoStat™ (version 2020).

## Results

Microplastic particles were detected in the feces of 21 dogs, while 6 showed no detectable particles. The mean concentration was 288.89 microplastic particles per gram, with variations observed among different sectors of the city. The northeastern and northwestern zones of the Santo Domingo canton exhibited the highest concentrations (means: 320 and 400 microplastic particles per gram, respectively), whereas the southeastern, southwestern, and peripheral areas showed lower values (means: 160, 250 and 200 microplastic particles per gram, respectively) (figure 1).

**Figure 1:**
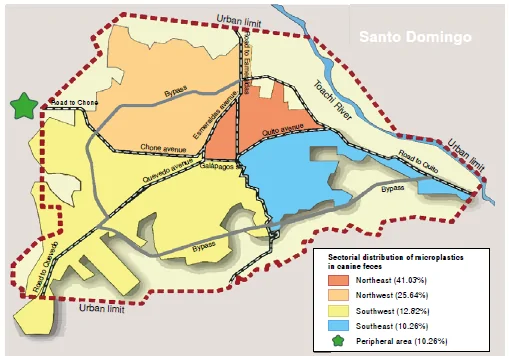
Sectoral distribution of the percentage of microplastics isolated from canine feces in the city of Santo Domingo

Morphological analysis using SEM, by comparison with controls, confirmed the presence of fiber-like structures (figure 2). No microplastic contaminants were observed in the blank samples. 

**Figure 2:**
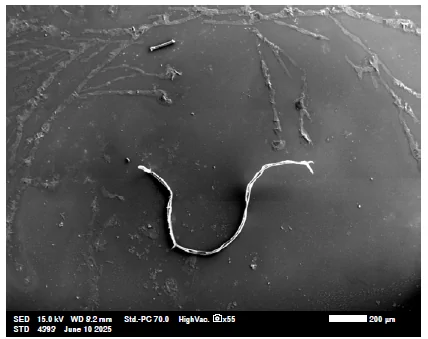
Microplastic fiber isolated from a canine fecal sample after potassium hydroxide digestion and observed under scainng electron microrscopy

Over a 30-day period, the daily generation of food-contact plastic waste was estimated, with an overall average of 30.21 g/person/day. Although variations were observed across sectors, correlation analysis showed no significant relationship between the amount of plastic waste generated and microplastic particles concentration in canine feces (p = 0.194), indicating that the results suggest a potential association rather than a direct causal relationship. 

Additionally, gastrointestinal diseases were reported in 16 households, while 11 showed no such conditions. Statistical testing between microplastics presence in canine feces and gastrointestinal disorders in human tutors revealed a significant association (p = 0.027; Cramer’s V = 0.463) of moderate strength (figure 3). These findings suggest a potential link between domestic exposure to microplastics and digestive health, without implying causality. 

**Figure 3:**
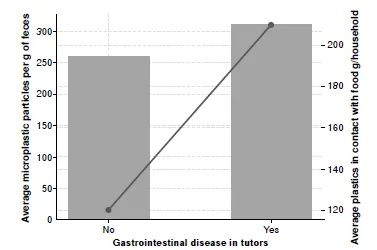
Relationship between the average concentration of microplastics in feces and the average household plastic waste generation, according to the presence of gastrointestinal disorders in the tutors

## Discussion

The average concentration of 288.89 microplastic particles per gram found in canine fecal samples collected from various sectors of Santo Domingo exceeds the range previously reported in pets. Zhang *et al.*^26^ reported concentrations equivalent to approximately 30 microplastic particles per gram, reinforcing the notion that companion animals reflect domestic microplastic exposure ^18^.

The geographic distribution revealed higher concentrations in the northwestern zone (400 microplastic particles per gram), followed by the northeastern (320 microplastic particles per gram), with lower levels in peripheral areas (≤ 200 microplastic particles per gram), likely influenced by differences in population density, plastic consumption, and waste management ^27^. Moreover, the highest generation of food-contact plastic waste coincided with areas of increased fecal microplastic load, suggesting that domestic environments are a significant source of exposure ^28^.

The statistically significant association (p = 0.027; Cramer's V = 0.463) between the presence of microplastics in pets and gastrointestinal diseases in their tutors aligns with human findings, where a higher fecal microplastics load has been documented in patients with inflammatory bowel disease ^29^^,^^30^. The applied methodology, based on KOH digestion, has proven to be highly sensitive and specific (> 90%), ensuring reliable recovery and identification of synthetic polymers ^5^^,^^24^.

Furthermore, dogs, due to their close coexistence with humans and their shared exposure to food, air, and household dust ^18^^,^^31^, emerge as useful sentinel organisms for estimating exposure to urban environmental contamination ^4^^,^^18^^,^^25^^,^^32^. Their physiology allows for the approximation of potential biological effects of microplastics in mammals, providing an indicative reference for assessing human health risk ^30^^,^^33^^,^^34^.

The findings of this study should be interpreted as exploratory associations rather than evidence of causality. The cross-sectional design allowed the identification of potential links between microplastics exposure and gastrointestinal health in humans and dogs, although it does not establish direct causal relationships.

The small sample size, convenience sampling, and lack of control for potential confounding variables limit the generalizability of the results. However, the primary objective was to document the presence of microplastics in the canton of Santo Domingo (Ecuador) rather than to evaluate demographic or lifestyle factors.

Despite methodological limitations, this work represents the first documented evidence of microplastics in canine feces in the canton, highlighting urban microplastic contamination and its potential shared impact on humans and animals. The findings underscore the value of dogs as bioindicators of environmental exposure and provide a strong foundation for future multidisciplinary research under the *One Health* approach, aimed at developing intersectoral preventive strategies.
